# Ankle strategies for step-aside movement during quiet standing

**DOI:** 10.1371/journal.pone.0281400

**Published:** 2023-03-07

**Authors:** Lingchao Xie, Sanghyun Cho

**Affiliations:** Department of Physical Therapy, Yonsei University, Wonju, South Korea; King Khalid University, INDIA

## Abstract

The mediolateral ankle strategy plays a crucial role in providing ankle stability in ground obstacle-avoidance behavior. This is achieved by changing basic walking patterns according to the characteristics of the obstacle. In daily life, it is more common to use step-aside movement (i.e., dodging step) for collision avoidance rather than side-stepping (i.e., widening standing base) when encountering an approaching pedestrian or bicycle. While studies have been conducted on the mediolateral ankle strategy contribution in obstacle avoidance using side-stepping, knowledge of step-aside movement is still inadequate. Therefore, we conducted an electromyography (EMG) analysis on the tibialis anterior (TA), peroneus longus (PL), and soleus (SOL) muscles, as well as measured center of pressure (CoP) displacement, and vertical ground reaction force (vGRF) of the standing leg, in order to understand the role of ankle muscles in step-aside movement during quiet standing. Fifteen healthy young men repeated twelve step-aside movements in both left and right directions. A Bayesian one-sample t-test was used to determine the sufficient step and participant counts. Multiple linear regression analysis was used to investigate the correlation between the muscle activity and CoP displacement or vGRF. The regression coefficients (β) of the left push phase and the right loading phase were tested against zero using a Bayesian one-sample t-test to identify the correlation between independent and dependent variables. We used the one-dimensional statistical parametric mapping (SPM1d) method to analyze the differences between and within the groups of EMG data based on the continuous time series. The results showed that the PL displayed a substantial contribution to the mediolateral ankle strategy during the push phase of step-aside movement, and also contributed to maintaining ankle stability during the loading phase. This suggested that screening for PL weakness and providing appropriate interventions and/or training approaches is especially critical for populations with walking stability problems.

## Introduction

Hoogkamer, Potocanac, and Duysens (2015) indicated that different leg movement adjustments for obstacle avoidance come with different risks, and side-stepping movement (i.e., widening standing base) showed a relatively low success rate [1]. Hof and Duysens (2018) revealed that when a leftward perturbing push force was applied to the trunk during the stance phases, tibialis anterior (TA), peroneus longus (PL), and soleus (SOL) muscle activations was observed [2]. This was consistent with van Leeuwen et al.’s suggestion that these muscles are mainly activated to displace the complementary center of pressure (CoP), thereby maintaining mediolateral gait stability [3]. This mediolateral ankle strategy plays a critical role in providing ankle stability in ground obstacle-avoidance behavior [2, 3].

However, obstacle avoidance is typically achieved through adaptive locomotor adjustments that change basic walking patterns according to the characteristics of the obstacle to be stepped over or the direction to be avoided [4, 5]. In daily life, it is more common to use step-aside movement (i.e., dodging step) for collision avoidance than side-stepping when encountering an approaching pedestrian, a bicycle, or tree in the walking path. Previous studies conducted on a treadmill have not investigated step-aside movements owing to the limitations of the equipment. Kondo et al. suggested that many older adults’ collison-avoidance behaviors are insufficient (i.e., frequent collision) or inefficient (i.e., exaggerated behavior) [6], which increases the risk of falls. According to a study by Robinovitch et al., the most common causes of falls among the older population are incorrect weight shifting and tripping. The activities associated with the highest proportion of falls were forward walking (24%, 54 of 227 falls), standing quietly (29 falls), and sitting down (28 falls) [7]. Therefore, understanding obstacle avoidance mechanisms and identifying an efficient functional training method to improve the ability of the older population to safely avoid obstacles is crucial. To date, a number of studies have investigated obstacle-avoidance behavior through step-over [8–10] and side-stepping movements [3, 11]; however, the mechanism of obstacle-avoidance behavior using step-aside movement remains unclear.

As prior studies have illustrated, the mediolateral ankle strategy is a potential compensatory strategy during steady-state walking, involving small but fast actions to shift the CoP relative to the center of mass (CoM) throughout the single stance phase [2, 3]. Therefore, before the subsequent foot placement, the CoM is controlled within the base of support (BoS) to prevent falling. This small but quick correction reduces the need for the next step of the contralateral foot [12, 13]. To further understand the role of the mediolateral ankle strategy in obstacle-avoidance behavior using step-aside movement, we investigated the correlation between independent variables (TA, PL, and SOL contraction) and dependent variables [CoP mediolateral (CoPx), anteroposterior (CoPy) displacement, and vertical ground reaction force (vGRF)]. Additionally, we chose to first analyze the step-aside movement during quiet standing and then analyze it during steady walking in a subsequent study. Because the analysis of step-aside movement during quiet standing was novel, we divided the movement into push and loading phases based on the principles of typical gait analysis description.

Previous studies have indicated that walking on a medially inclined ramp with an elevated lateral part of the foot increases PL contraction [14] and ankle muscle contractions induce CoP displacement [15]. Based on this, we hypothesized that 1) in the pushing foot of step-aside movement, PL contraction will move the CoP medially, and SOL contraction will move the CoP anteriorly and contribute to vGRF generation. 2) In the loading foot, the PL will contribute the most to prevent excessive ankle inversion. Therefore, the aim of the present study was to determine the ankle strategy and mechanism of step-aside movement, which can help in understanding and/or providing appropriate interventions for individuals with decreased gait stability, particularly older adults, to help prevent falls and injuries.

## Materials and methods

### Participants

Healthy men aged 18–28 years were recruited as participants for the study. Owing to the size of the foot insole sensors, the average foot size of the participants was between 260 and 270 cm. Anyone who had balance issues, pes planus, chronic ankle instability [16], or previous major lower extremity problems were excluded. The initial sample size was ten; thus, we continued to recruit participants until the number reached a meaningful evidence threshold, using the Bayesian sequential sampling method [17]. In total, 15 healthy participants [24 ± 2 yrs; 1.75 ± 0.05 m; 76 ± 5 kg; mean ± standard deviation (SD)] completed the experiment. Prior to each test session, each participant provided written informed consent, and the Yonsei University Mirae Institutional Review Board (approval no. 1041849-202203-BM-050-04) approved this study.

### Procedure

The most comfortable and natural step width for a human is close to the shoulder width [18, 19]. Thus, participants were asked to perform step-aside movements with their natural step widths at a comfortable speed. According to the definition in the relevant literature, foot placement is actively adjusted by the central nervous system at the time of gait initiation [20]. Since the purpose of this study was not to confirm this, foot placement was fixed to one step-width level. We applied three parallel lines of white pinstripe tape on the floor at shoulder-width distances as the step-aside guidelines. Participants looked ahead and obtained visual feedback on the step width by looking at a mirror placed in front of them.

**Fig 1:**
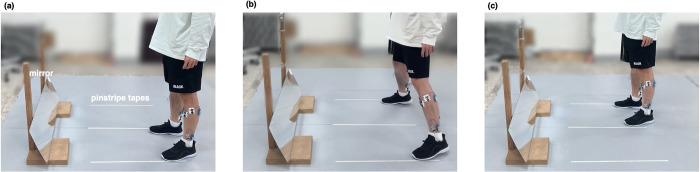
Experimental setup and procedure for the step-aside movement.

**Fig 2:**
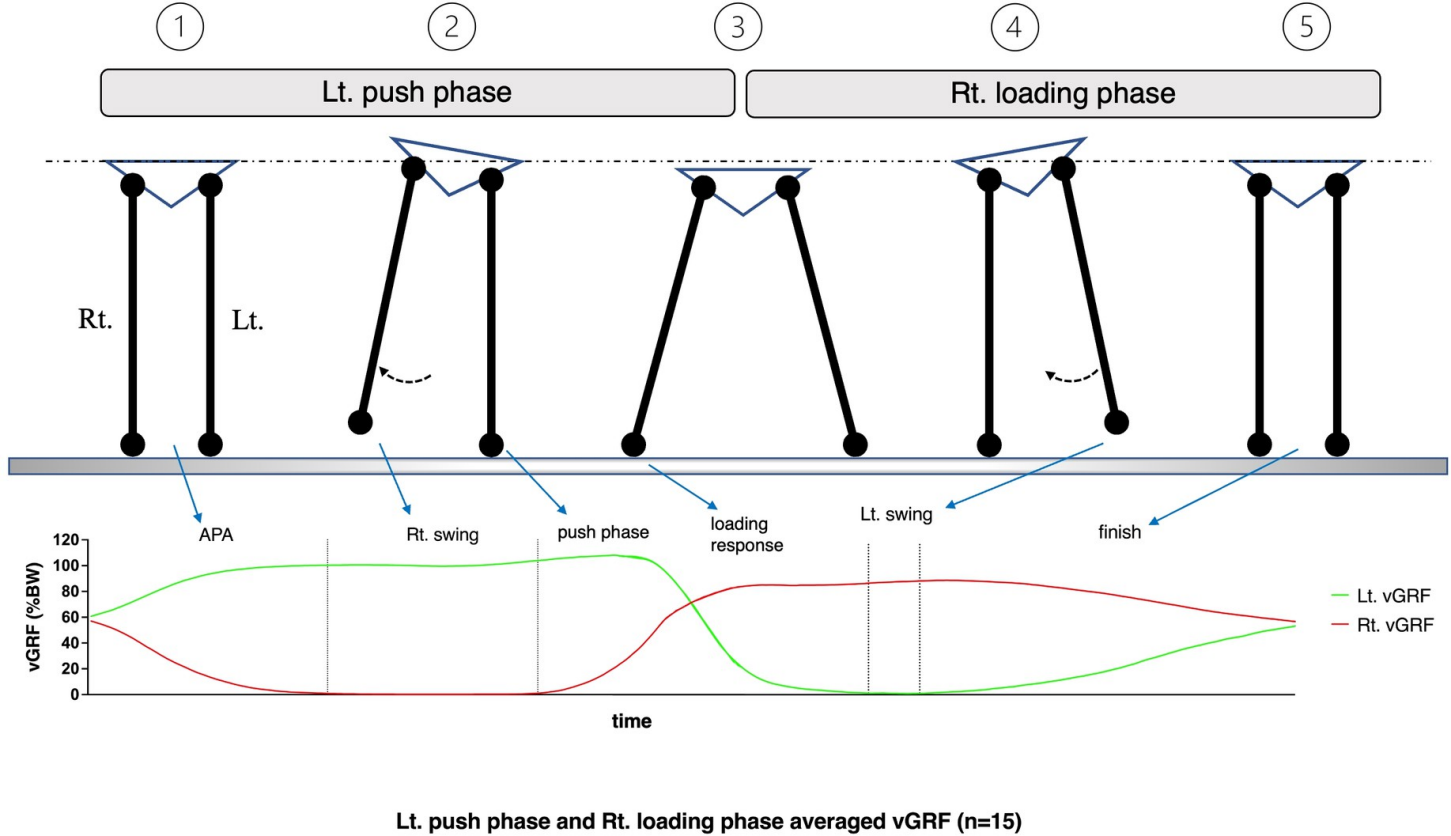
“Step-aside to the right” movement sequence was divided into two phases: The “left push” phase and the “right loading” phase. According to the method by Yiou et al., each phase was further divided into three periods [20].

In practice sessions for step-aside movements, all participants were asked to stand quietly on the left and middle guidelines, lift their right foot, and move it to the right guideline. Afterwards, they were asked to lift their left foot, move it to the middle guideline, and finally return to the quiet standing position (a to c in Fig 1, 1 to 5 in Fig 2). After 3 s of rest, step-aside movement to the left was performed. As indicated by Wu et al. (2014), there is an ever-present variability in motor execution [21]. Therefore, the participants were asked to perform two practice sessions before the experiment to become accustomed to their most comfortable movement speed. After being fully equipped and practiced, participants repeated the movements 12 times in each direction for a total of 24 steps. Data from the first and last steps in each direction were not included in the statistical analysis. The determination of the step count is explained in detail in the data analysis section.

### Data acquisition and signal processing

Noraxon Ultium (Noraxon Inc., Scottsdale, AZ, USA) wireless surface electromyography (EMG) sensors were used to record bilateral muscle activity from the PL, which provides foot eversion and plantarflexion, TA, which provides dorsiflexion and inversion, and SOL, which provides plantarflexion and inversion [13]. The recording electrodes were placed 20 mm apart with respect to the muscle fiber to be measured, as recommended by the SENIAM [22]. Prior to electrode attachment, the skin was prepared by shaving and alcohol swapping. The raw signals were recorded and processed using MR3.18^®^ software (Noraxon Inc., Scottsdale, AZ, USA). EMG data were recorded at a sampling frequency of 2000 Hz, high-pass filtered at 20 Hz, rectified, and low-pass filtered at 50 Hz, following the protocol by Rankin et al. [23]. The sampling rates of the CoP and vGRF data were 500 Hz. The start and end points of a single step-aside movement are illustrated in the phase and period division sections. For amplitude normalization, the filtered-rectified muscle EMG data of a single step-aside movement were divided by the average of all step-aside movement muscle EMG data in the trial. Time was normalized to 200 data points.

Participants wore a pair of knitted shoes (Natso Knit Jogging Shoes, KeonJong, S-Korea) equipped with the Noraxon Ultium Insole, and total foot pressure (vGRF) and foot CoP displacement data were collected. An increase in CoP displacement represented a shift to the right (medial side of the left foot and lateral side of the right foot) and anterior sides. A decrease in the CoP displacement value indicated the opposite. We conducted a statistical analysis of the differential CoP movement (change in CoP from the initial position) in the mediolateral or anteroposterior direction. CoP displacement was obtained by subtracting the first value from the CoP value of the average report for each participant [15]. vGRF data were normalized to the body weight of each participant.

### Phase and period division

**Fig 3:**
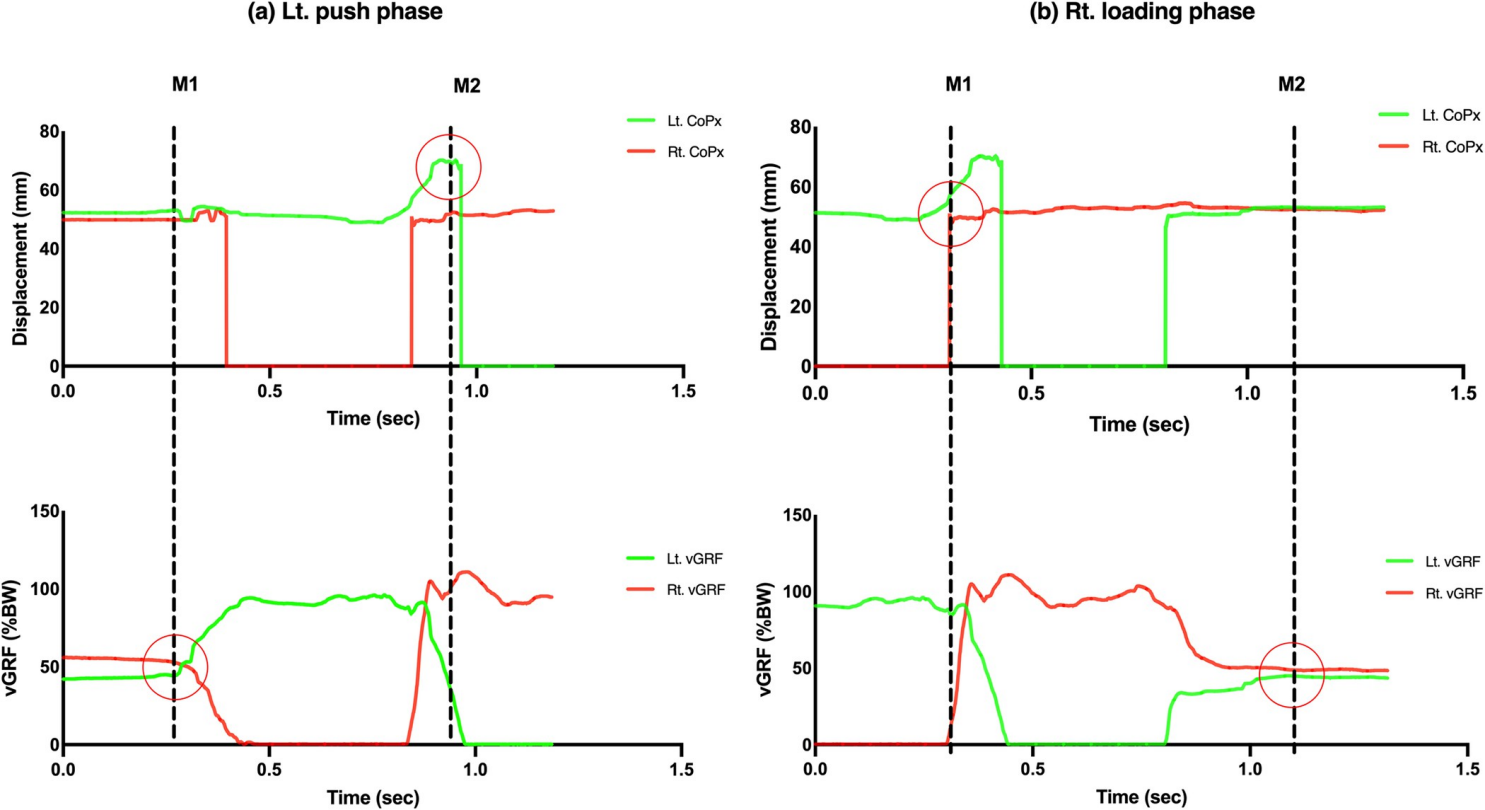
Marking example of the start (M1) and end (M2) point for a single (a) “left push phase” and a single (b) “right loading phase”. Red circles indicate the moment for M1 and M2. CoPx: center of pressure mediolateral displacement; vGRF: vertical ground reaction force.

**Fig 4:**
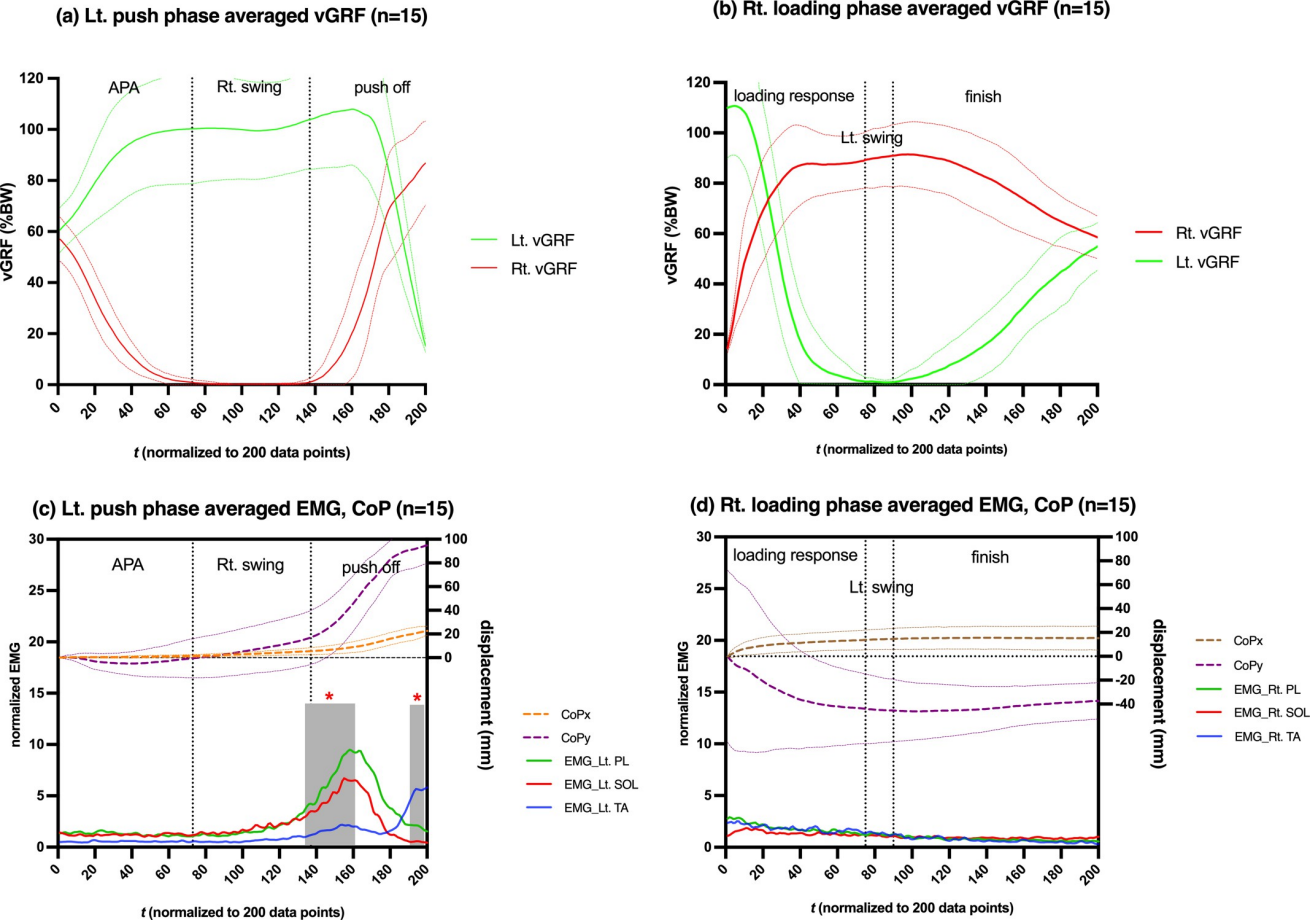
The change in (a,b) bilateral vGRF, and (c,d) ipsilateral normalized EMG amplitudes and CoP displacement during the step-aside movement. Data on the plot represent the means from 15 subjects. SDs are not shown on EMG amplitudes for clarity purposes. Gray boxes on (c) indicate statistically significant different regions between EMG activations from SPM1d analysis. *: p < 0.05. Two vertical dotted lines divide each phase into three periods, respectively. APA: anticipatory postural adjustment.

Based on the vGRF and CoP displacement of both feet, we defined the left push phase as the period from the time when the right foot vGRF decreased below 50% of the body weight (BW) (Fig 3A, M1) to the time when the left foot lifted (Fig 3A, M2). The right loading phase was defined as the period from right foot landing (Fig 3B, M1) to the time when vGRF of both feet was 50% of the BW (Fig 3B, M2). Each phase was divided into three periods, following the method described by Yiou et al. [20] (Fig 2). The left push phase has the following stages: 1. anticipatory postural adjustment (APA), from quiet standing to right foot lift; 2. the right swing period, from right foot lift to right foot loading, and 3. the push-off period, from right foot loading to left foot lift (Figs 2 and 4C). The right loading phase has the following stages: 1. the loading response period, corresponding to the previous push-off period; 2. the left swing period, from left foot lift to left foot loading; and 3. the finish period, from left foot lift to quiet standing (Figs 2 and 4D). In total, ten left push and right loading phases were marked in one trial dataset per participant.

The average activation pattern report for each participant was made separately for ten left push and ten right loading phases, with the time normalized to 200 data points to obtain high data resolution and better results in multiple regression.

### Statistical analysis

#### Bayesian statistical analysis

To determine the number of step-aside movement repetitions, we analyzed the relationship between the CoPx displacement and EMG amplitudes of the left PL (EMG_Lt.PL) at each step in the left push phase and extracted the regression coefficient (β) data to represent the step number. The Bayesian sequential sampling method revealed that the value of BF_10_ displayed “very strong evidence” when the total step count was > 8. Therefore, excluding the first and last steps, we decided to collect data from the participants who repeated the step-aside movement ten times in each direction. The number of participants was also determined using the same method; only β was obtained by analyzing the average data from ten step-aside movements for each participant. We examined the absolute values of the regression coefficients between CoPx displacement and the three muscles using the Bayesian sequential sampling method, and the value of BF_10_ showed “extreme evidence” when the number of participants reached 15 [17]. We compared β values between CoP displacement and muscle activations in the left and right push phases using a Bayesian paired samples t-test, which showed no significant difference. Accordingly, we determined that only the step-aside to the right movement (left push and right loading phases) would be analyzed in the subsequent data analysis. Normal distribution was confirmed using the Shapiro–Wilk test before conducting the Bayesian one-sample t-test. All multiple linear regression and Bayesian statistical analyses were conducted using JASP (version 0.16.2.0; JASP Team, 2022).

Additionally, the β of each phase was tested against zero separately using a Bayesian one-sample t-test to determine the correlation between independent and dependent variables in the step-aside movement.

#### One-dimensional statistical parametric mapping (SPM1d)

SPM1d is a continuous data analysis method that is more practical for muscle activity analysis with one-dimensional (1D) time-varying characteristics. Unlike traditional statistical methods that analyze the differences in peak or mean values, this method can analyze the differences in multi-muscle EMG data based on continuous time series [24]. In this study, the differences in TA, PL, and SOL contractions between and within the groups were tested by statistically examining the entire EMG time series using SPM1d. All SPM analyses were implemented in Python 3.8 (www.python.org) using the open-source software package SPM1d 0.4.8 [25] (www.spm1d.org).

One-way analysis of variance (ANOVA) was used to determine the time period with significant differences in three muscle activations during the left push or right loading phases, and the post-hoc paired t-test was used to assess the significance between muscle activations during that time period. An SPM{F} or SPM{t} was created by calculating the conventional univariate t- or F-statistic at each point of the muscle activation [26]. We also employed SPM Hotelling’s T-square test, which is the vector field equivalent to the two-sample t-test [27, 28], to check the magnitude and time period of the push-loading differences. The magnitude at each individual time point was calculated separately using the scalar output statistic, SPM{T^2^}. SPM{F}, SPM{t}, and SPM{T^2^} statistics indicated the magnitude of the differences. To test the null hypothesis (H0), we calculated the critical threshold at which only α % (5%) of the smooth random curves would be expected to traverse. This approach is based on the Random Field Theory [29] and has been validated for 1D data [30, 31].

#### Multiple linear regression analysis

The β values of all variables in the model with the greatest coefficient of determination (R^2^) were extracted, and those with a multicollinearity problem or significance level of p > 0.05 were treated as missing values for further statistical analyses.

The following regression analysis was performed to infer the relationship between muscle activations and subsequent CoP displacement and vGRF:

$$CoPx=\beta_{TA}∙{EMG}_{TA}+\beta_{PL}∙{EMG}_{PL}+\beta_{SOL}∙{EMG}_{SOL}+{error}_{m}$$

$$CoPy=\beta_{TA}∙{EMG}_{TA}+\beta_{PL}∙{EMG}_{PL}+\beta_{SOL}∙{EMG}_{SOL}+{error}_{m}$$

$$vGRF=\beta_{TA}∙{EMG}_{TA}+\beta_{PL}∙{EMG}_{PL}+\beta_{SOL}∙{EMG}_{SOL}+{error}_{m}$$

## Results

### Left push phase EMG

As shown in Fig 4, for the first 2/3 of the left push phase (APA and rightswing period), the BW moved toward the left foot without much ankle muscle activation. For the last 1/3 of the left push phase (push-off period), the ankle muscles were activated, resulting in peak vGRF, and then the BW moved towards the right foot. When the left vGRF increased beyond 100% BW (125.0–173.0 data points), EMG_Lt.PL and EMG_Rt.SOL also reached their peaks to enable the push off. SPM1d’s one-way ANOVA indicated significant differences (p < 0.001) among the ankle muscle activations during 134.1–160.5 and 193.0–199.0 data points (Fig 4C, gray boxes).

During the last 1/20 of the left push phase (193.0–199.0 data points), vGRF decreased rapidly, and the roles of the three ankle muscles changed. EMG_Lt.PL and EMG_Lt.SOL hit their lowest points, whereas EMG_Lt.TA peaked (Fig 4A and 4C). The results of SPM1d’s one-way ANOVA post hoc analysis revealed statistically significant differences (p < 0.001) among the three ankle muscle EMG amplitudes.

In the left push phase, the left foot average CoP displacement was 22.59 ± 3.98 mm (mean ± SD) medially and 99.66 ± 15.84 mm anteriorly (Fig 4C). Notably, the SD of the CoP displacement data for the 15 participants during the last 180.0–200.0 data points was rather small.

### Right loading phase EMG

For the first 1/10 of the right loading phase (0–20.0 data points, initial foot contact),the right vGRF increased rapidly, but only small ankle muscle contractions were observed. Then, muscle activation continued to decrease, and only a few muscle activations were observed even if the right vGRF reached higher than 80% BW during 29.0–146.0 data points. The results of SPM1d’s one-way ANOVA also did not find any significant differences among the three ankle muscle EMG amplitudes.

In the right loading phase, the right foot average CoP displacement was 15.56 ± 10.01 mm laterally and 46.14 mm ± 55.09 posteriorly (Fig 4D). However, the SD of the CoPy displacement data during the 0–20.0 data points was rather large.

### Comparison between push and loading phase EMG

**Fig 5:**
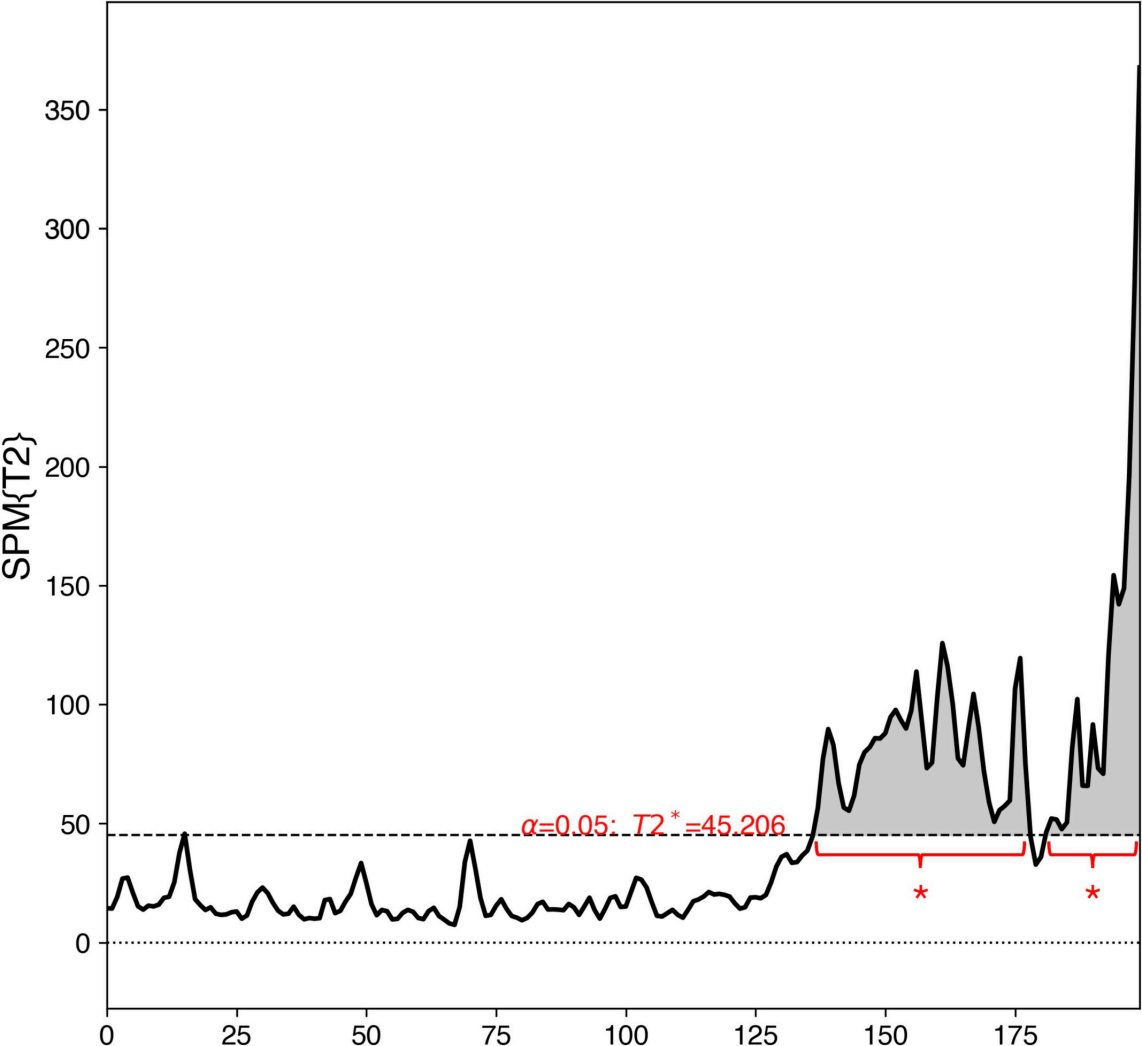
SPM results (Hotelling’s T^2^ test statistic trajectory) depicting the differences in muscle activities between the left push and the right loading phases. The critical threshold (black dashed line) is 45.206. *: p < 0.001.

Fig 4 shows that the coordination patterns of the ankle muscles in the left push and right loading phases were different. We used SPM1d Hotelling’s T^2^ test to investigate the magnitude of the difference. As illustrated in Fig 5, there were two major regions (136.0–178.0 and 180.9–199.0 data points) of the T^2^ trajectory (a supra-threshold cluster-shaded region) exceeding the critical threshold, which demonstrated statistically significant differences (p < 0.001) in the coordination pattern of ankle muscle activations between the two phases.

### Left push phase multiple regression analysis

**Fig 6:**
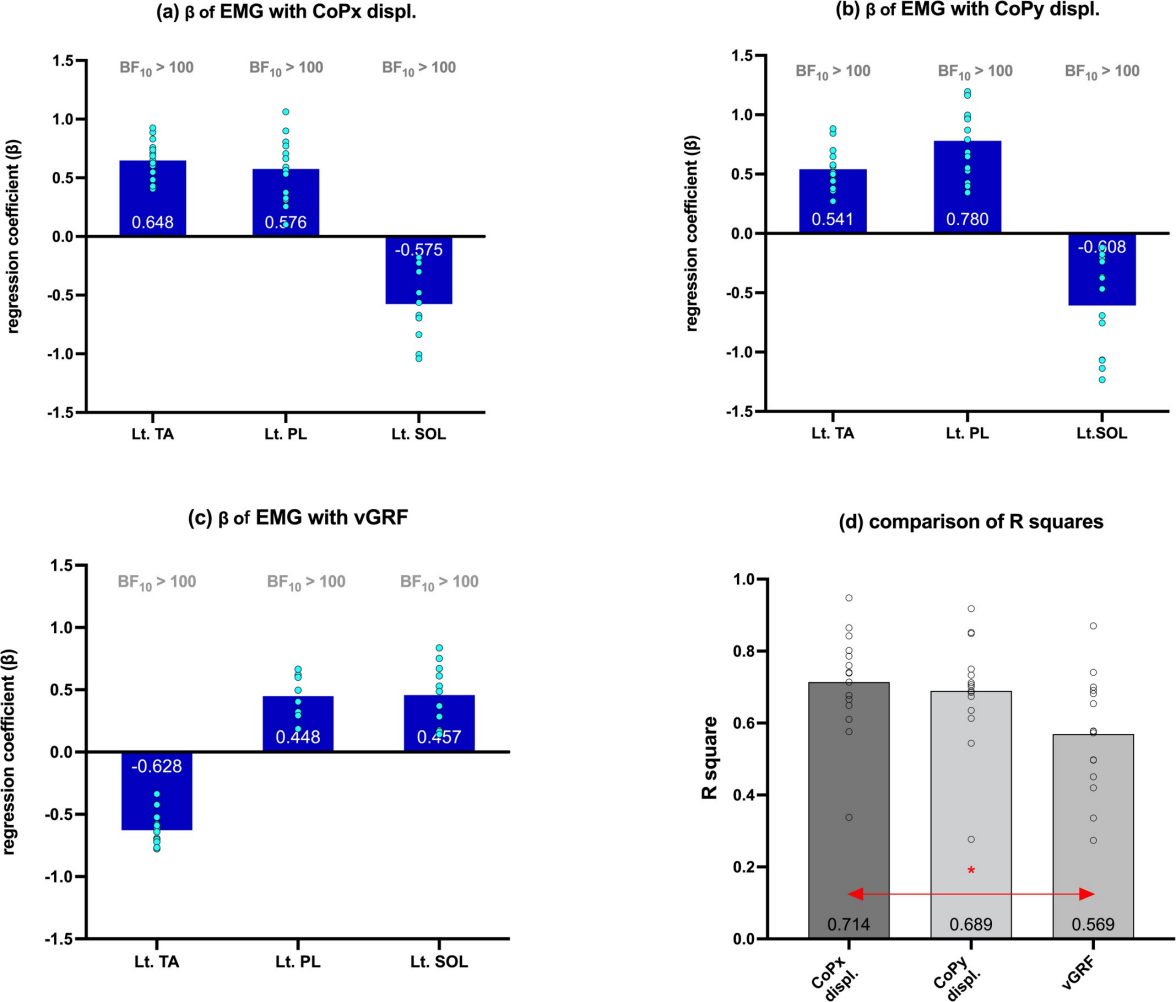
Standardized regression coefficients (β) of EMG_Lt.PL, EMG_Lt.TA, and EMG_Lt.SOL with (a) foot CoPx displacement, (b) foot CoPy displacement, and (c) vGRF during the left push phase. The box height represents the mean value, and the circles represent individual data points. The Bayes factors (BF_10_) indicate the evidence level that the regression coefficients are different from zero. (d) R^2^ of the three dependent variables Red arrow with (*) shows the significantly different pair on ANOVA post-hoc comparison (p < 0.05 Tukey). Numbers in the box represent the mean values of the variable.

**Fig 7:**
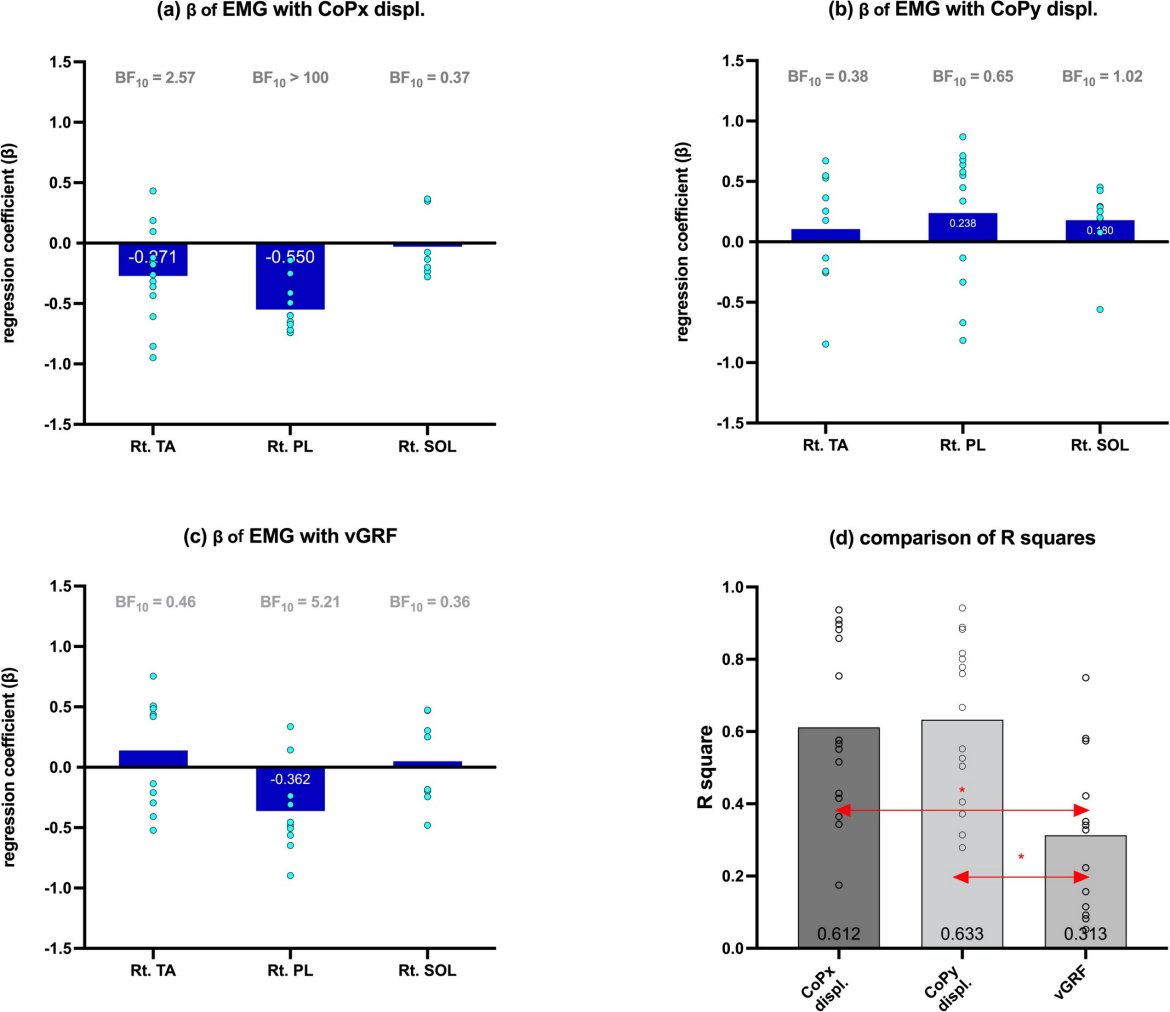
Standardized regression coefficients (β) of EMG_Lt.PL, EMG_Lt.TA, and EMG_Lt.SOL with (a) foot CoPx displacement, (b) foot CoPy displacement, and (c) vGRF during the right loading phase. The box height represents the mean value, and the circles represent individual data points. The Bayes factors (BF_10_) indicate the evidence level that the regression coefficients are different from zero. (d) R^2^ of the three dependent variables Red arrow with (*) shows the significantly different pair on ANOVA post-hoc comparison (p < 0.05 Tukey). Numbers in the box represent the mean values of the variable.

Three ankle muscle EMG amplitudes were designated as the independent variables for multiple linear regression analysis. Figs 6 and 7 summarize all the R^2^ values and standardized regression coefficients (β). All β values in the Figures were statistically significant (p < 0.05) and were free from multicollinearity. We used a Bayesian one-sample t-test to examine H0. All absolute values of the average β of EMG_Lt.TA, EMG_Lt. PL, and EMG_Lt.SOL were within 0.408–0.780. All of their BF_10_ values were greater than 100, which meant “extreme evidence” to reject H0 (p < 0.05). ANOVA post-hoc comparison revealed that there was no significant difference between the average R^2^ in CoPx (0.714) and CoPy (0.689) displacement. However, the difference between R^2^ in CoPx displacement and vGRF (0.569) was statistically significant (Fig 6D, p < 0.05 Tukey).

### Right loading phase multiple regression analysis

In the right loading phase, the absolute values of the average β of EMG_Rt.TA, EMG_Rt. PL, and EMG_Rt.SOL were within 0.030–0.550. The Bayesian one-sample t-test showed “extreme evidence” (BF_10_ > 100) and “moderate evidence” (10 > BF_10_ > 3) only when testing the β values of EMG_Rt.PL with CoPx displacement and vGRF against zero, respectively. Therefore, H0 was rejected at a significance level of p < 0.05 (Fig 7). ANOVA post-hoc comparison indicated no significant difference between the average R^2^ in CoPx (0.612) and CoPy (0.633) displacement. However, there were significant differences between R^2^ in CoPx, CoPy displacement, and vGRF (0.313) (Fig 7D, p < 0.05 Tukey).

## Discussion

Step-aside movement is a common type of obstacle-avoidance behavior, and understanding its mechanism can facilitate the development of functional training approaches to reduce the risk of falls during obstacle avoidance. The purpose of this study was to provide a preliminary understanding of the ankle strategy and mechanism of step-aside movement during quiet standing and prepare for subsequent research on straight walking and the development of functional training approaches. To simplify the understanding, we introduced a new phase division method to divide the step-aside movement into push and loading phases, and then used two new statistical approaches in the field of biomechanics, Bayesian statistics and SPM1d, to analyze the mechanism of step-aside movement.

### Phase division

As shown in Fig 3, we divided the step-aside movement into “push phase” and “loading phase” based on the moment when the left foot lifted from the floor and the right foot initiated contact with the floor. The results showed that this division method could clearly distinguish between the two phases (Fig 4). In addition, the SPM Hotelling’s T^2^ test compared the muscle activations in these two phases and revealed two regions with strong statistical significance (p < 0.001) (Fig 5), further validating this division method.

### Left foot CoP trajectory

In the left push phase, we found that, in line with the findings of Yiou et al., the CoPy moved backward (17.06 ± 4.918 mm) to promote the initial forward propulsive force during the APA period (Fig 4C), which was consistent with normal gait initiation [20]. The mediolateral ankle strategy involves the CoPx displacement of the ipsilateral foot during the contralateral foot swing phase [2, 20]. In the left push phase, we found that CoPx displacement was 3.978 ± 1.181 mm, which indicated the presence of a mediolateral ankle strategy in the step-aside movement. The left foot CoP trajectory showed a small SD (Fig 4C), suggesting distinct medial and anterior displacements in the left push phase.

### Left ankle muscle contribution

Consistent with previous studies, our regression analysis indicated that medial displacement of the CoP during the left push phase was mainly caused by PL contraction, which induced ankle eversion [2, 3, 15]. Although PL and SOL are both plantarflexors, the regression analysis showed that activations of these two muscles resulted in anterior and posterior CoP displacements, respectively (Fig 6). Anatomically, this may be due to the different insertion points of the muscles [32]. PL contraction produces eversion, and plantarflexion pulls the base of the first metatarsal inferiorly [33], thereby moving the CoP anteriorly. Cimadoro et al. found that if the body segment leans forward, causing the CoM to be located anterior to the ankle joint, the SOL contracts to pull the body backward, thus maintaining the CoM within the BoS [34].Similar to other previous studies [35, 36], plantarflexion caused by SOL contraction displaced the CoP posteriorly in the present study.

During the last 1/20 of the left push phase, ankle muscle activations were significantly different (p < 0.001) from each other, leading to different impacts on CoP displacement. Regression analysis revealed that TA resulted in medial and anterior CoP displacement, contrary to our hypothesis based on Kim et al. (2003) [15]. In the present study, the push-off action generated by PL contraction first induced CoP displacement towards the medial anterior direction, and the TA contraction-produced toe clearance action may have accelerated it, which led to contradicting statistical results.

SPM1d’s one-way ANOVA post-hoc analysis revealed that EMG_Lt.PL and EMG_Lt.SOL were significantly larger than EMG_Lt.TA almost simultaneously when the vGRF exceeded 100% BW (125.0–173.0 data points, p < 0.001). EMG_Lt.TA was significantly larger than EMG_Lt.PL and EMG_Lt.SOL (193.0–199.0 data points, p < 0.001) almost simultaneously when the vGRF rapidly decreased. Therefore, during the left push phase, we presumed that both EMG_Lt.PL and EMG_Lt.SOL contributed similarly to the vGRF increase, and EMG_Lt.TA produced toe clearance action and a vGRF decrease.

Extreme evidence of statistical significance (BF_10_ > 100) was found among the β values of all the three muscle activations and vGRF (Fig 6C), demonstrating that PL and SOL activation resulted in vGRF exceeding 100% BW and that TA activation subsequently contributed to the rapid decline of vGRF to complete toe clearance. However, the relatively small R^2^ value suggested that other factors affected the increase in vGRF, such as the body weight shift and CoM acceleration or deceleration [37]. One-way ANOVA showed that R^2^ for CoPx displacement was significantly greater (p < 0.05) than that for vGRF; however, no significant difference was found with R^2^ for CoPy displacement (Fig 6D), which demonstrated that ankle muscles contributed more to CoP displacement than to vGRF.

Overall, these results suggest that all three ankle muscles played a very important role in the push phase of the step-aside movement. PL and SOL contributed to push-off, implying that individuals with ankle muscle weakness may experience limitations in safely completing the step-aside push-off movement. In addition, in an emergency obstacle-avoidance situation involving step-aside movements, falls and/or injuries to the ankle joint may occur. Previous studies have also indicated a relationship between PL weakness and recurrent ankle sprains [38].

Second, TA contributed the most to completing toe clearance. As reported by Perera et al., a weakened TA may lead to a reduction in minimum toe clearance, thereby increasing the probability of falling or tripping [39]. Furthermore, PL contraction that was found simultaneously with TA contraction was thought to control inversion and maintain balance [40], further highlighting the importance of PL.

### Right foot CoP trajectory

The average CoPx displacement with a small SD demonstrated an explicit lateral displacement during the right loading phase in the step-aside movement. However, the large SD of CoPy displacement illustrated the uncertainty of the loading point. This may be because the initial floor contact point changed during each step-aside movement. Nevertheless, the SD gradually decreased in the subsequent time, and the average CoP trajectory tended to level off around the 110^th^ data point, indicating that the CoP displacement of the 15 participants would eventually return to a position near the starting point of the left push phase.

### Right ankle muscle contribution

The initial loading action can be divided by the floor contact location into the fore foot strike (tip-toeing), mid-foot strike, and rear foot strike [20], resulting in a large deviation of the right-foot CoPy displacement during the 0–20.0 data points. Owing to the different landing points, the muscle activations differed. Fore foot strike induces PL activation to maintain the concavity of the foot during tip-toeing, as described in Gray’s Anatomy [41]. In the present study, although no statistical difference was found between any two muscles during the right loading phase, a certain activity of the PL can be seen during the 0–20.0 data points (Fig 4D). The Bayesian one-sample t-test indicated extreme evidence of statistical significance (BF_10_ > 100) for the regression coefficients (β) between EMG_Rt.PL and CoPx displacement, and moderate evidence (BF_10_ = 5.21) between EMG_Rt.PL and vGRF (Fig 7A and 7C). When the rear foot strike occurs first, according to the normal gait mechanism, the TA may cause eccentric contraction to produce resistance to the initiating plantarflexion [42]. However, in this study, all statistical analyses failed to detect the contribution of the TA in the right loading phase, even though some activations could be seen during the 0–20.0 data points.

The PL contracts to ensure ankle stability during the pre-landing phase [16, 43]. Although our testing protocol could not provide sufficient instability elements to the ankle joint and only a weak EMG–Rt.PL can be seen in Fig 4D, the results were adequate to support the evidence for a PL-dominated mediolateral ankle strategy [2]. Patients with chronic ankle instability (CAI) reportedly tend to have reduced PL contraction before foot-ground contact and show differences in neuromuscular control compared to those without CAI [16, 44, 45]. Therefore, we presumed that the ankle muscles play a role in ankle stabilization only during the initial foot contact, even without any significant difference among the three ankle muscle activities.

In summary, the PL displayed a substantial contribution not only in the push phase of the step-aside movement, but also in maintaining ankle stability during the loading phase, making PL weakness screening and providing appropriate intervention and/or training approaches for individuals with decreased gait stability even more critical.

### Study limitations

This study has several limitations. One limitation is that the data were collected in a laboratory setting, which may not accurately reflect step-aside movements in everyday life. Additionally, there is a lack of previous research on step-aside movements, which prevents us from comparing the results of this study with those of other studies. This may have limited the significance of the findings. Finally, the movement in this study was relatively simple and planned in advance by the participants, which may not accurately represent typical obstacle-avoidance behaviors. In future research, we plan to investigate step-aside movements during normal walking to address these limitations.

## Conclusion

Our study conclusions are as follows:

1. The PL-dominated mediolateral ankle strategy was more apparent in the left push phase. The mechanism of CoPy backward displacement during the APA period in the step-aside movement was parallel to normal gait initiation.
2. The PL and TA contributed the most to push-off and toe-clearance during the push phase, respectively.
3. Ankle muscles, especially the PL, play a role in stabilization only during initial foot contact in the right loading phase.
4. Based on the above statements, screening for PL weakness and providing appropriate interventions and/or training approaches is especially critical for populations with walking stability problems.
